# Dimensions of sexual self-concept and sexual dysfunctions in middle-aged men: results of the Bavarian Men’s Health-Study

**DOI:** 10.1038/s41443-025-01119-1

**Published:** 2025-07-04

**Authors:** Valentin H. Meissner, Victoria A. Söhne, Andreas Dinkel, Matthias Jahnen, Stefan Schiele, Martina Kron, Jürgen E. Gschwend, Kathleen Herkommer

**Affiliations:** 1 Technical University of Munich, School of Medicine and Health, Department of Urology, TUM University Hospital, Munich, Germany; 2 Technical University of Munich, School of Medicine and Health, Department of Psychosomatic Medicine and Psychotherapy, TUM University Hospital, Munich, Germany; 3 Institute of Epidemiology and Medical Biometrics, University of Ulm, Ulm, Germany

**Keywords:** Sexual dysfunction, Risk factors

## Abstract

Sexual self-concept is multidimensional and a key construct in understanding people’s sexuality. The etiology as well as the relationship and causal direction between its different dimensions and sexual dysfunctions are not fully understood. A population-based cohort (*N* = 5665) of 50-year-old men completed questionnaires on dimensions of sexual self-concept (toughness, body image, sexual self-esteem, and perceived pressure with regard to sexuality) and sexual dysfunctions. Associations were assessed using multivariable linear regression analyses. Erectile dysfunction (ED), lifelong/acquired and subjective/variable premature ejaculation (PE), and low libido (LL) were associated with a more negative body image (*p* < 0.001), lower sexual self-esteem (*p* < 0.001), and higher perceived pressure with regard to sexuality (*p* < 0.001). ED, lifelong/acquired and subjective/variable PE were associated with toughness (*p* < 0.001), an association with LL could not be shown. A negative sexual self-concept was independently associated with sexual dysfunctions in middle-aged men highlighting that sexual self-concept represents a relevant factor in the biopsychosocial conception of sexual dysfunctions. The causal direction of this relationship needs to be determined in longitudinal studies.

## Introduction

Self-concept is a multidimensional construct, containing different aspects of a person’s self-perception. Main aspects of the self are ‘the psychological self, the social self, the familial self, the sexual self, and the coping self’ [1]. Sexual self-concept is a developmental construct that can evolve with experience, socialization, and psychological changes. It is a person’s perception of themselves as a sexual being and therefore a key construct to understand people’s sexuality [2, 3]. However, a clear definition and standardized measurement tools are lacking, while many different dimensions and facets are known [4]. As Deutsch and colleagues pointed out, sexual self-concept is a broad model, and there is low consensus for what factors belong to the model [4]. Recently, a review of 67 studies found that researchers investigated 34 different dimensions pertaining to the sexual self-concept [2]. The broad nature of the concept allows to include various variables that touch on the self-perception as a sexual human being. Variables that were most frequently studied are sexual self-esteem, sexual assertiveness, sexual self-efficacy, and body image. However, a few studies also looked at sexual fear of sexual social comparison [2]. In the current study, four specific dimensions of sexual self-concept were of interest (i.e., toughness, body image, sexual self-esteem, and perceived pressure with regard to sexuality) assuming that these dimensions might be especially relevant in the context of sexual behavior and sexual dysfunctions.

Masculine norms such as toughness, restrictive emotionality or anti-femininity are related to poor mental health and men’s health behaviors more broadly [5]. Since masculine norms are also partly defined by sexual functioning, adherence to these norms may hinder men’s willingness to seek help when sexual dysfunctions arise.

Body image is another frequently assessed dimension of sexual self-concept. Men’s self-rated attractiveness has been reported higher than women’s [6], still, prevalence of body dissatisfaction ranged from 9.0–28.4% in an US sample of men [7]. Further important dimensions of sexual self-concept include sexual self-esteem, which was described as the “tendency to positively evaluate one’s capacity to sexually relate to a partner” [8]. This includes feeling confident in sexual encounters, rating one’s sexual skills highly, and considering oneself a good sexual partner [8]. Sexual performance anxiety is defined as an individual’s excessive concern about their ability to satisfy a sexual partner [9]. While an association between sexual performance anxiety and erectile dysfunction (ED) has been established, the causal direction is not known and may be bidirectional [9, 10]. This applies also for other sexual dysfunctions such as premature ejaculation (PE) and low libido (LL) [10]. High levels of sexual performance anxiety might result in elevated levels of perceived pressure with regard to sexual performance. While the relationship between sexual dysfunctions and sexual performance anxiety is well known, far less research has addressed perceived pressure with regard to sexuality in the context of sexual dysfunctions.

ED, PE, and LL are reported as the most common male sexual dysfunctions and pathogenesis is regarded as multidimensional [11–13]. In addition to physical risk factors such as age, diabetes mellitus, dyslipidaemia, hypertension, cardio-vascular disease, obesity, metabolic syndrome, lack of exercise, smoking and drug use for example, psychological factors play an important role in the etiology and understanding of these dysfunctions. For instance, different dimensions of self-concept such as self-perceived likeability were associated with sexual functioning in a previous study [14]. Negative attitudes towards different aspects of one’s body, e.g. towards muscularity, were associated with higher body self-consciousness during physical intimacy [15]. More negative body image facets were related to ED [16, 17] and PE [16], but there was no association found between body image and libido [18, 19]. However, among those with decreased sexual interest, more than 10% named not feeling attractive as a cause [19]. The importance attributed to each construct of masculinity did not differ between men with and without ED [20]. Reduced self-confidence, generally and in sexual encounters, as well as fear about inadequate sexual performance were commonly mentioned concerns in men with PE [21]. Both lower sexual self-esteem and higher perceived pressure with regard to sexual performance were positively correlated with severity of PE [22].

Knowledge on the relationship between sexual self-concept and sexual dysfunctions is sparse, and available research with large population-based samples is mostly limited to women [23, 24]. Moreover, longitudinal research is lacking and the causal direction of the relationship is unknown, but a reciprocal relationship might be possible. Hence, the aim of this study was to investigate the relationship between sexual self-concept and male sexual dysfunctions in a large population-based sample of 50-year old men. Since numerous variables are known to capture the multifaceted concept of sexual self-concept, the current study focused on four different dimensions of sexual self-concept (i.e., toughness, body image, sexual self-esteem, and perceived pressure with regard to sexuality) and their associations with the three most common male sexual dysfunctions ED, PE, and LL.

## Materials and methods

### Study population and procedure

The study population consists of men who were included in the Bavarian Men’s Health-Study (BMH-Study) between April 2020 and March 2023. The BMH-Study is an ongoing German study which focuses on various aspects of male physical, mental and sexual health in a large, population-based sample of 50-year old Bavarian men concomitantly participating in a prostate cancer screening trial. Men were invited by simple random sampling from local population registers within 100 km of the study center. At three time points, including baseline, 5 years, and 10 years after baseline, participants visit the clinical center for an interview and a brief physical examination, where they complete standardized questionnaires [25]. Men who completed the questions about at least one dimension of sexual self-concept as well as the questions about at least one sexual dysfunction were included in this analysis. To avoid restrictions, no further exclusion criteria were applied. Hence, 5665 out of 5855 (96.8%) men were evaluated. Taking missing data in further variables into account, 4538 (80.1%, toughness), 4538 (80.1%, body image), 4355 (76.9%, sexual self-esteem), and 4423 (78.1%, perceived pressure with regard to sexuality) men, respectively, were available for the final regression analyses. The study was approved by the ethical review committee of the Technical University of Munich. All men were fully informed about the study procedure and provided written informed consent before their enrolment. All methods were performed in accordance with the relevant guidelines and regulations.

### Participants and measures

#### Sexual self-concept

Four dimensions of sexual self-concept (i.e., toughness, body image, sexual self-esteem, and perceived pressure with regard to sexuality) were addressed. These four dimensions were selected because they might be especially relevant in the context of sexual behavior and sexual dysfunctions. To reduce respondent’s burden in this multi-topic survey, shortened versions of available questionnaires were used in some cases. At the time of the conception of the study, no established German questionnaires assessing sexual self-esteem and perceived pressure with regard to sexuality were available. Therefore, the study team consensually defined new items to measure these variables (i.e., three items for sexual self-esteem and four items for perceived pressure with regard to sexuality). The items were constructed in German. To present an English version of the items that were originally developed in German, two bilingual researchers independently translated the items to English. The translations were compared, and differences were discussed until a final version was reached. This version was independently back-translated by two other bilingual researchers. The result showed high agreement with the original item wording.

*Toughness* was assessed using three items from the Toughness Norm subscale of the German version of the Male Role Norm Scale (MRNS) [26, 27]. The items consisted of the following statements: “When a man is feeling a little pain he should try not to let it show very much.”, “Nobody respects a man very much who frequently talks about his worries, fears, and problems.”, and “I think a young man should try to become physically tough, even if he’s not big.”. Participants were asked to rate their attitude towards masculinity on a 5-point Likert scale (1 = fully disagree to 5 = fully agree). The three items were among the highest loading items of the subscale [26]. So, a higher score represents higher toughness and an understanding of a more traditional male role norm, namely the expectation that men should be tough and self-reliant, whereas a lower score represents lower toughness. Cronbach’s α was 0.67.

*Body image* was assessed using three items from the Dresden Body Image Inventory (DKB-35) which potentially relate to sexual performance [28, 29]. The items consisted of the following statements: “I like my body”, “I enjoy showing my body” and “I have pleasant and intense feelings of my body in sexuality”. The participants were required to rate each item on a 5-point Likert scale ranging from 1 (not at all) to 5 (completely). Higher scores represent a more positive body image. Cronbach’s α was 0.68.

*Sexual self-esteem* was assessed using three items, which were newly designed for this study: “During sex, I am a good lover”, “During sex, I pay attention to my partner’s desires”, and “During sex, I am imaginative”. The participants rated each item on a 5-point Likert scale (1 = not at all to 5 = completely). Higher scores represent higher sexual self-esteem. Cronbach’s α was 0.71.

*Perceived pressure with regard to sexuality* was assessed using four newly designed items: “I often panic about living up to the expectations of being a man during sex”, “I have the impression that nowadays, too much is expected from men during sex”, “With regards to sexuality, I feel put under pressure”, and “I worry whether I correspond to the public image of a ‘real man’”. The participants were asked to rate each item on a 5-point Likert scale (1 = not at all to 5 = completely). Higher scores represent higher perceived pressure with regard to sexuality. Cronbach’s α was 0.76.

#### Sociodemographic, psychosocial and lifestyle factors

A self-report list was used to assess sociodemographic variables, including being in a partnership (yes vs. no), having children (0 vs. 1 vs. ≥2), and level of education (low vs. intermediate vs. high). Anxiety and depression were assessed using the German version of the two-question screening tool Generalized Anxiety Disorder-2 (GAD-2) and the Patient Health Questionnaire-2 (PHQ-2) [30]. Both measures are rated on a four-point Likert scale (0–3). A summary score ≥3 indicates clinical levels of anxiety and depression, respectively. These measures have proven reliable and valid in previous studies [30]. Body height and weight were measured and Body Mass Index was calculated and categorized (<25 kg/m^2^ vs. 25–<30 kg/m^2^ vs. ≥30 kg/m^2^).

#### Erectile dysfunction

The presence of ED was assessed using the German version of the IIEF-EF [31, 32] and the Erection Hardness Score (EHS) [33]. The severity of ED was classified as mild (IIEF-EF score 22–25), mild to moderate (IIEF-EF score 17–21), moderate (IIEF-EF score 11–16), or severe (IIEF-EF score 6–10) [34]. Since men with failed sexual intercourse, few sexual attempts or non-heterosexual orientation identity are not suitable for assessment by the IIEF-EF domain, the EHS was additionally asked [31, 35]. The EHS is a validated single-item patient-reported outcome (PRO) of erection hardness ranging from 0 (penis does not enlarge) to 4 (completely hard and fully rigid) [33]. The presence of ED was defined as an IIEF-EF score ≤25 or an EHS score ≤3 [31, 33].

#### Premature ejaculation

PE was assessed using two items of the Sexual Complaints Screener for Men (SCS-M) [36], an evidence-based screening tool that assesses men’s sexual complaints in the past six months. The SCS-M was developed based on expert opinion to assess sexual problems in a medical practice setting, and its validity and reliability were recently established in Turkish men [37]. Participants were divided into three groups according to the International Society for Sexual Medicine Ad Hoc Committee for the Definition of Premature Ejaculation [38] (no PE vs. lifelong/acquired PE vs. variable/subjective PE). The screening measure consisted of the following two items.

(I). “Some men cannot control their sexual excitement so that they cum (ejaculate) before or shortly (within approximately 2 min) after penetration. Has this happened to you during the last 6 months?” Response options were “no sexual activity”; “never/almost never”; “rarely”; “sometimes”; “often”; “almost all the time/almost always”.
(II). “Has this been a personal problem for you?” Here, response options were “not at all a problem”; “a very small problem”; “some problem”; “a considerable problem”; “a very great problem”.

They were identified as having “no PE”, if they answered the first question with “never/almost never” and the second question with “not at all a problem”. In contrast, participants were identified as having lifelong/acquired PE when answering the first question with “often” or “almost all the time/almost always” and the second question with “some problem”, “a considerable problem” or “a very great problem”. If participants answered the first question with “never/almost never”, “rarely” or “sometimes” and the second question with “some problem”, “a considerable problem” or “a very great problem” there were identified as having variable/subjective PE.

#### Low libido

The presence of LL was assessed with the question “How often have you felt sexual desire during the past 4 weeks?” (very frequently, frequently, occasionally, rarely, very rarely/never), which was adapted from a study of men and women in Germany [12]. The latter two response options were defined as LL.

### Statistical analysis

Descriptive statistics were used to summarize participant characteristics. Multivariable linear regression analyses with backward elimination (elimination level of 5%) were used for each dimension of sexual self-concept to identify associations with sexual dysfunctions, adjusting for other explanatory factors. Results were reported in terms of estimates (beta), along with 95% confidence intervals (CI). All statistical tests were two-sided, exploratory and performed at the 0.05 level of significance. Data analyses were conducted using the Statistical Analysis System (SAS), version 9.4 (SAS Institute Inc., Cary, NC, USA).

## Results

**Fig. 1:**
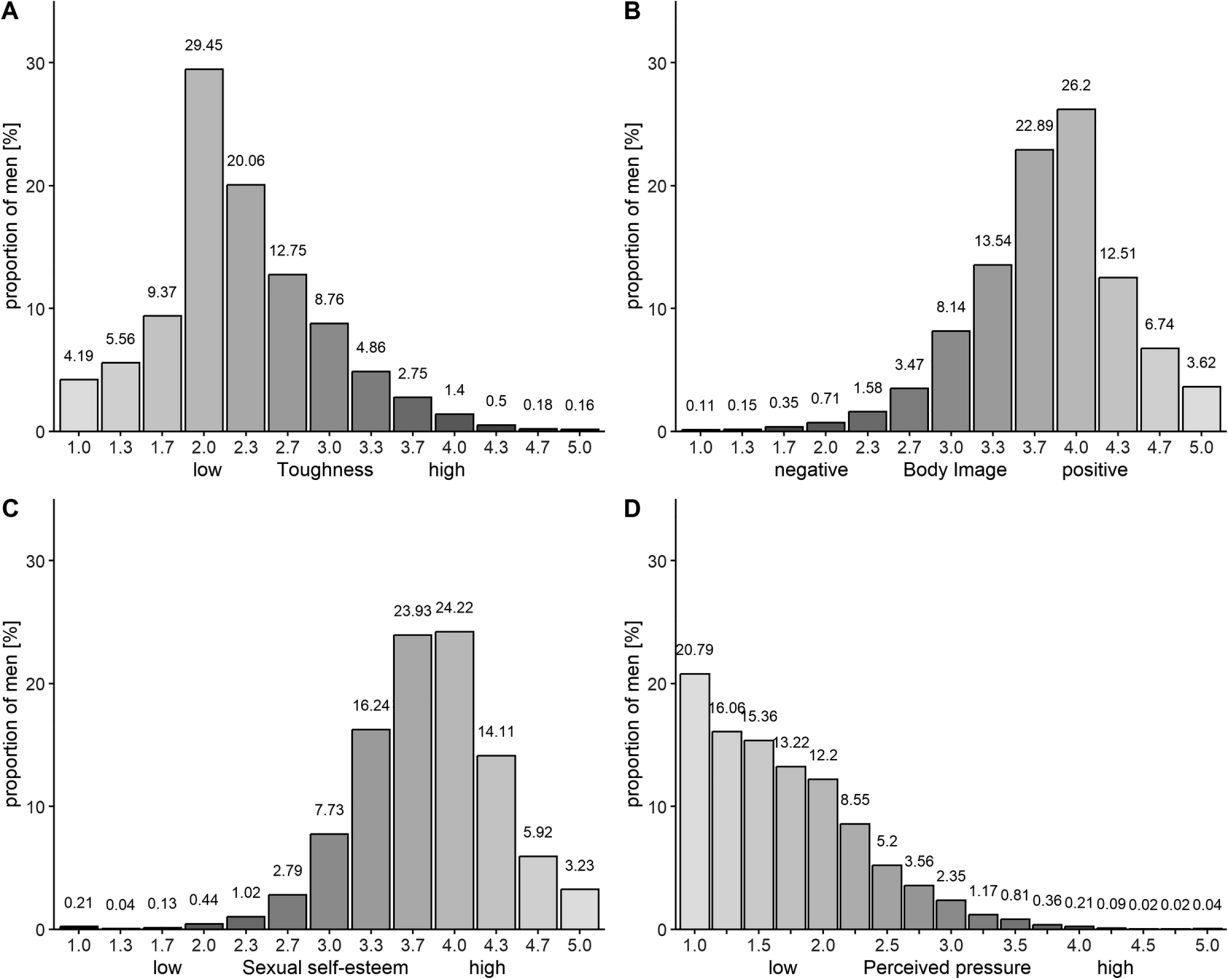
Distribution of men concerning the four dimensions of sexual self-concept. **A** shows the frequency distribution of the mean values of the dimension toughness, **B** body image, **C** sexual self-esteem, and **D** perceived pressure with regard to sexuality.

**Fig. 2:**
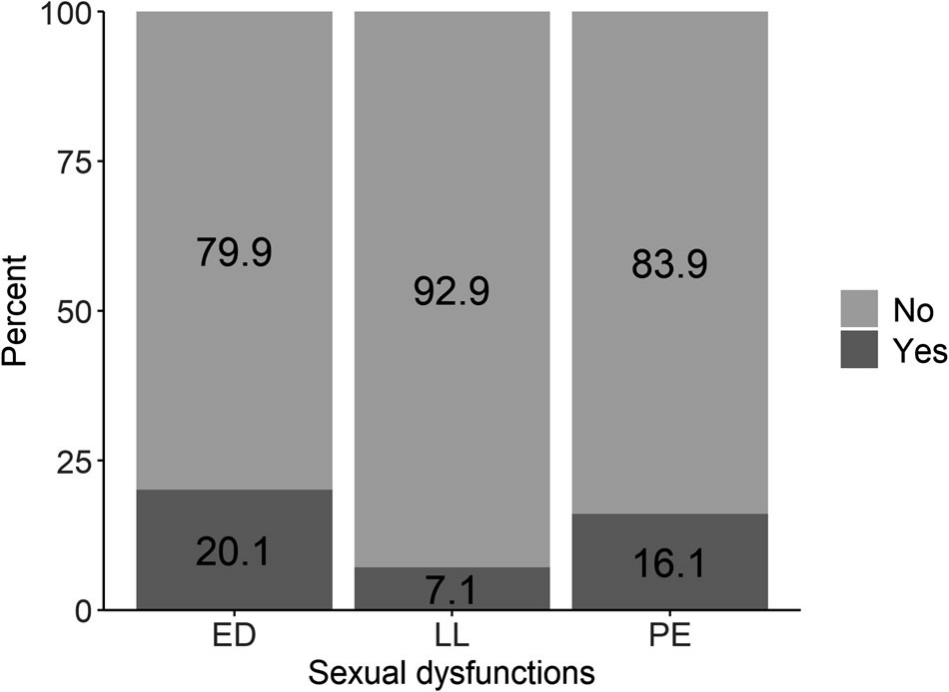
Distribution of the 3 assessed sexual dysfunctions erectile dysfunction (ED), low libido (LL), and premature ejaculation (PE).

A total of 5665 men were included in this analysis. A detailed description of baseline characteristics is presented in Table 1. Fig. 1 shows the frequency distribution of the mean values of men with respect to the four different dimensions of sexual self-concept (A–D, Fig. 1). With regard to sexual dysfunctions, 20.1% of men had ED, 6.0% reported lifelong/acquired PE, 10.1% subjective/variable PE, and 7.1% reported LL (Fig. 2).

**Table 1:** Baseline characteristics of the study sample (*n* = 5665).

Sexual self-concept		
Toughness, mean (SD)		2.29 (0.7)
Body image, mean (SD)		3.77 (0.6)
Sexual self-esteem, mean (SD)		3.78 (0.6)
Perceived pressure with regard to sexuality, mean (SD)		1.70 (0.6)
**Sexual dysfunctions**		
Erectile dysfunction		
No	4441	79.9
Yes (IIEF-EF score ≤25 and EHS score ≤3)	1120	20.1
Premature ejaculation		
No	4058	83.9
Yes (lifelong/acquired)	291	6.0
Yes (subjective/variable)	489	10.1
Low libido		
No	5152	92.9
Yes	391	7.1
**Sociodemographic factors**		
Partnership		
No	744	13.3
Yes	4458	86.7
Children		
0	1700	30.0
1	1129	19.9
≥2	2836	50.1
Level of education		
low	551	9.7
intermediate	1109	19.6
high	4005	70.7
**Psychosocial factors**		
Depression (PHQ-2)		
<3	5350	95.1
≥3	277	4.9
Anxiety (GAD-2)		
<3	5346	95.1
≥3	278	4.9
**Lifestyle factors**		
Body Mass Index (kg/m^2^)		
<25	2035	36.2
25–<30	2548	45.3
≥30	1041	18.5

Percents refer to among the observed data and do not include missing data. All numbers are sample sizes n (%) except when reported otherwise.

*IIEF-EF* international index of erectile function-erectile function, *EHS* erection hardness score, *PHQ-2* patient health questionnaire-2, *GAD-2* generalized anxiety disorder-2, *SD* standard deviation.

Concerning the three sexual dysfunctions, the multivariable linear regression analyses revealed that ED (*p* < 0.001), lifelong/acquired PE (*p* < 0.001), and subjective/variable PE (*p* < 0.001) were associated with higher toughness, an association with LL could not be shown. ED (*p* < 0.001), lifelong/acquired PE (*p* < 0.001), subjective/variable PE (*p* < 0.001) and LL (*p* < 0.001) were associated with a more negative body image. ED (*p* < 0.001), lifelong/acquired PE (*p* < 0.001), subjective/variable PE (*p* < 0.001), and LL (*p* < 0.001) were associated with lower sexual self-esteem. ED (*p* < 0.001), lifelong/acquired PE (*p* < 0.001), subjective/variable PE (*p* < 0.001), and LL (*p* < 0.001) were associated with higher perceived pressure with regard to sexuality. Among these associations, further sociodemographic, lifestyle, and psychosocial variables showed significant associations with the four dimensions of sexual self-concept (Table 2).

**Table 2:** Multivariable linear regression analyses with backward elimination of the four dimensions of sexual self-concept.

	Toughness			Body image			Sexual self-esteem			Perceived pressure		
	beta	95% CI	*p*	beta	95% CI	*p*	beta	95% CI	*p*	beta	95% CI	*p*
Sexual dysfunctions												
Erectile dysfunction (y vs. n)	0.09	0.04; 0.14	<0.001	−0.21	−0.25; −0.17	<0.001	−0.20	−0.24; −0.15	<0.001	0.52	0.48; 0.56	<0.001
Premature ejaculation			<0.001			<0.001			<0.001			<0.001
Lifelong/acquired premature ejaculation (y vs. n)	0.12	0.05; 0.20		−0.24	−0.31; −0.18		−0.40	−0.46; −0.33		0.42	0.35; 0.48	
Subjective/variable premature ejaculation (y vs. n)	0.08	0.02; 0.14		−0.16	−0.21; −0.11		−0.17	−0.22; −0.12		0.30	0.25; 0.35	
Low libido (y vs. n)	—	—	—	−0.25	−0.32; −0.18	<0.001	−0.32	−0.39; −0.24	<0.001	0.15	0.07; 0.22	<0.001
Sociodemographic factors												
Partnership (n vs. y)	0.17	0.10; 0.24	<0.001	−0.12	−0.18; −0.06	<0.001	—	—	—	0.16	0.10; 0.22	<0.001
Children (n vs. y)	—	—	—	−0.06	−0.09; −0.02	0.003	−0.04	−0.07; −0.00	0.030	0.11	0.07; 0.15	<0.001
Level of education			0.005			0.003			0.002			0.019
Level of education (high vs. low)	−0.07	−0.14; −0.01		−0.08	−0.14; −0.03		−0.09	−0.14; −0.03		−0.01	−0.06; 0.04	
Level of education (intermediate vs. low)	−0.01	−0.08; 0.07		−0.04	−0.10; 0.02		−0.04	−0.11; 0.02		0.05	−0.01; 0.11	
Psychosocial factors												
Depression (PHQ-2) (y vs. n)	0.22	0.11; 0.33	<0.001	−0.25	−0.34; −0.16	<0.001	−0.10	−0.18; −0.02	0.014	—	—	—
Anxiety (GAD-2) (y vs. n)	0.20	0.09; 0.30	<0.001	−0.24	−0.32; −0.15	<0.001	—	—	—	0.26	0.18; 0.33	<0.001
Lifestyle factors												
BMI			<0.001			<0.001			—			0.013
BMI (<25 vs. 25—<30)	−0.03	−0.07; 0.01		0.17	0.13; 0.20		—	—	—	−0.03	−0.06; 0.01	
BMI (≥30 vs. 25–<30)	0.09	0.04; 0.14		−0.28	−0.32; −0.23		—	—	—	0.04	−0.00; 0.08	

*CI* confidence interval, *PHQ-2* patient health questionnaire-2, *GAD-2* generalized anxiety disorder-2, *BMI* body mass index.

## Discussion

By analyzing data of more than 5000 50-year-old men, the current study showed that a specific facet of the self-concept, namely sexual self-concept, was also associated with functional sexual problems. Results of the current study revealed that a more negative sexual self-concept was independently associated with one or more of the three most common sexual dysfunctions.

Every fifth man in the current study had ED, defined as an International Index of Erectile Function (IIEF)-EF score ≤25 and an Erection Hardness Score ≤3. This integrative definition has been previously used to include all men independent of sexual activity and sexual orientation identity [25], since the sole use of the IIEF does not capture men without sexual intercourse, few sexual attempts or non-heterosexual orientation identity [31]. Results of a recently published US-American study of more than 17,000 men showed a comparable prevalence of ED (20%) in 50-year-old prostate cancer patients [39]. In this analysis, 6.0% of men reported lifelong/acquired PE, and the prevalence of subjective/variable PE was nearly twice as high (10.1%). The prevalence rate of lifelong/acquired PE in this study is comparable with results from other studies (i.e., 6.2 and 8.0%, respectively) [40, 41] using the same ISSM evidence-based definition of PE [38]. Notably, subjective/variable PE was higher in these studies compared to results of this study (14 and 18%, respectively vs. 10.1%). This might be partly explained by the fact that in these studies, PE was assessed in Turkey and China and there has been reported a strong cultural influence on the prevalence of subjective PE [42]. However, the mean age of the study samples in the current study, was higher than the study population of the Turkish and Chinese study (50 years vs. 42 and 34 years, respectively). Since age is associated with a delay in ejaculation latency and more sexual experience, this difference may be also responsible for the discrepancy between the prevalence rates of subjective/variable PE between the Turkish and Chinese studies and the current study.

Finally, 7.1% of the men in our sample reported LL which is in line with results from other large nationally representative studies in men at the same age from Denmark and Germany, respectively (7.7 and 4.2%) [12, 43].

Men who rated being physically strong, not talking about their problems, and not showing pain as important factors of masculinity were classified as tough and that they adhere to traditional masculine norms. Interestingly, most men in this study did not agree to the toughness statements and presented therefore a low level of toughness. This stands in line with results of the multinational Men’s Attitudes to Life Events and Sexuality (MALES) study reporting on divergent perceptions of masculinity. For instance, being honorable, self-reliant, and respected by friends were important attributes of masculinity. Conversely, factors stereotypically associated with toughness or masculinity, e.g. being physically attractive and sexually active were deemed to be less important to men’s sense of masculinity [20]. Further, the ability to perform sexually can be an important factor for traditional thinking and understanding of toughness and masculinity [44]. Consistently, ED and PE, both aspects of sexual performance, were associated with higher scores of toughness.

Body image is considered a multidimensional construct, and previous studies have shown that a negative image of one’s body and physical appearance is quite common [45, 46]. In the current analysis, most men had an overall positive body image. However, having at least one of the three investigated sexual dysfunctions was associated with a more negative body image. These findings stand in line with results from a small online community sample of gay and bisexual men. Negative behavioral body image in sexual situations and evaluative body dissatisfaction were associated with PE. A lower affective body esteem and an increased drive for muscularity were associated with ED [18]. Data from a larger study on sexual function of 367 men (military personnel) age 40 or younger revealed that a more negative male genital self-image was associated with ED [17].

In the current study lower sexual self-esteem was associated with all of the three assessed sexual dysfunctions. These results stand in line with a previous study which found that men with PE experience more fear of failure in sexual situations and worry more about the satisfaction of their partner during sexual intercourse than men without PE [47]. Recent studies showed an association of low sexual self-confidence with low libido [48, 49]. The authors hypothesized that men prioritize their sexual performance over their own satisfaction [50], which could increase their libido and sexual interest. Likewise, perceived attractiveness of one’s partner was reported to be an important factor for male sexual interest [19], which might explain the association between LL and a lower sexual self-esteem found in the current analysis.

Higher perceived pressure with regard to sexuality was associated with ED, PE and LL. To our knowledge, no previous study has investigated this particular dimension of sexual self-concept in the context of sexual dysfunctions, although in times of modern media people might easily be confronted with unrealistic ideals possibly aggravating the perception of pressure. A higher perception of pressure and its impact on psychological distress could be a risk factor for sexual dysfunctions. Sexual anxiety, assessed with items inquiring about anxiety during sexual activity and worrying about performance, was associated with ED [17]. On the other hand, results showed that men with ED are afraid of ridicule due to their inability to perform adequately in sexual encounters [21] or are embarrassed about their sexual problems [22]. This might lead to a greater perception of pressure.

Special attention should be given to the fact that subjective and variable PE, which is in general distinguished from lifelong/acquired PE and not seen as a medical disease, showed the same results as lifelong/acquired PE. To our knowledge, this is the first study to investigate a possible relationship of subjective/variable PE with sexual self-concept, since this special population is normally not assessed. More importantly, associations with subjective/variable PE were seen for all four dimensions of sexual self-concept. Therefore, it is crucial to regard these men at risk for an increased psychological burden and they should be treated in the same way as men with lifelong/acquired PE.

This is the first study investigating four different dimensions of sexual self-concept and the three most common sexual dysfunctions in a large population-based sample of more than 5000 men. Additionally, all men were 50 years old, which provides a good generalizability. Sexual dysfunctions (i.e., ED and PE) were assessed using validated questionnaires and contrary to other studies that recruited participants on selected websites and collected data via online-surveys, this study comprises a sample of men who were randomly invited via mail to take part in a prostate cancer screening trial. Therefore, the current sample might include more health-concerned men resulting in a recruitment bias. Although LL was not assessed by a validated questionnaire, the question was taken from a large population-based study. Furthermore, sexual dysfunctions and dimensions of sexual self-concept are subjective and self-reported and might therefore be at risk for biased reporting. The cross-sectional design of this study does not allow causal conclusions to be drawn, therefore further longitudinal analyses are needed to determine whether sexual dysfunctions at a younger age are associated with a more negative sexual self-concept later in life, or vice versa. Finally, in order to limit participant’s burden in the assessment of various variables as part of a large screening trial, we decided to use very short scales for the assessment of the different dimensions of sexual self-concept. The scales were made up of selected items from existing self-report measures or were newly constructed. These short scales were not validated before. Therefore, our results should be replicated in further studies.

## Conclusion

A more negative sexual self-concept was independently associated with all three sexual dysfunctions in middle-aged men. Importantly, a negative sexual self-concept was associated not only with lifelong/acquired PE, but also with subjective/variable PE. Therefore, affected men with subjective/variable PE perceive a comparable psychological burden and should therefore be seen as men with PE, at least from a psychological point of view. These results provide further insights into sexual health among middle-aged men and underline that sexual self-concept is not only related to psychological factors and emotional well-being, but also to somatic and medical factors such as sexual dysfunctions. Whether a negative sexual self-concept triggers sexual dysfunctions or vice versa has to be determined in further longitudinal studies.

## Data Availability

All original data are available upon reasonable personal request to valentin.meissner@tum.de.
